# Prenatal metal exposure in the Middle East: imprint of war in deciduous teeth of children

**DOI:** 10.1007/s10661-016-5491-0

**Published:** 2016-08-05

**Authors:** M. Savabieasfahani, S. Sadik Ali, R. Bacho, O. Savabi, M. Alsabbak

**Affiliations:** 1 P.O. Box 7038 Ann Arbor, MI USA; 2 Department of Obstetrics and Gynecology, Basra Maternity Hospital, Basra Medical School, P.O. Box 1633 Basra, Iraq; 3 Department of Pediatric Dentistry, School of Dentistry, Lebanese University in Beirut, Beirut, Lebanon; 4 Dental Research Center, School of Dentistry, Isfahan University of Medical Sciences, Isfahan, Iran

**Keywords:** Metal exposure, Deciduous teeth, Lead, Iraq, Lebanon, Iran, War-related pollution

## Abstract

In war zones, the explosion of bombs, bullets, and other ammunition releases multiple neurotoxicants into the environment. The Middle East is currently the site of heavy environmental disruption by massive bombardments. A very large number of US military bases, which release highly toxic environmental contaminants, have also been erected since 2003. Current knowledge supports the hypothesis that war-created pollution is a major cause of rising birth defects and cancers in Iraq. We created elemental bio-imaging of trace elements in deciduous teeth of children with birth defects from Iraq. Healthy and naturally shed teeth from Lebanon and Iran were also analyzed for trace elements. Lead (Pb) was highest in teeth from children with birth defects who donated their teeth from Basra, Iraq (mean 0.73–16.74 ^208^Pb/^43^Ca ppm, *n* = 3). Pb in healthy Lebanese and Iranian teeth were 0.038–0.382 ^208^Pb/^43^Ca ppm (*n* = 4) and 0.041–0.31 ^208^Pb/^43^Ca ppm (*n* = 2), respectively. Our hypothesis that increased war activity coincides with increased metal levels in deciduous teeth is confirmed by this research. Lead levels were similar in Lebanese and Iranian deciduous teeth. Deciduous teeth from Iraqi children with birth defects had remarkably higher levels of Pb. Two Iraqi teeth had four times more Pb, and one tooth had as much as 50 times more Pb than samples from Lebanon and Iran.

## Introduction

A pandemic of developmental neurotoxicity affects millions of children worldwide (Grandjean and Landrigan 2014). Prenatal exposures to industrial chemicals, particularly lead (Pb), are among known causes of this pandemic. Given the seriousness of this global health issue, accurate measurement of prenatal exposures has become a scientific priority. Deciduous teeth originate in fetal life and may prove useful in measuring prenatal metal exposures.

In war zones, the explosion of bombs, bullets, and other ammunition releases multiple neurotoxicants into the environment, adding to the burden of childhood exposures. Recent studies in Iraq indicate widespread public exposure to neurotoxic metals (Pb and mercury) accompanied by unprecedented increases in birth defects and cancers in a number of cities (Savabieasfahani 2013). Current knowledge supports the hypothesis that war-created pollution is a major factor in the rising numbers of birth defects and cancers in Iraq.

The Middle East has been the site of a massive environmental disruption by bombardments. In 2015 alone, the USA dropped over 23,000 bombs in the Middle East. Twenty-two thousand bombs were dropped on Iraq/Syria (Zenko 2016). US military bases also produce and release highly toxic environmental pollutants in the Middle East. Though our knowledge is limited, a recent report by Physicians for Social Responsibility (PSR) offers a conservative estimate of two million killed in the Middle East since the 2003 US invasion of Iraq. Around one million people have been killed in Iraq, 220,000 in Afghanistan, and 80,000 in Pakistan. A total of around 1.3 million, not included in this figure, have been killed in other recently created war zones such as Yemen and Syria (Physicians for Social Responsibility (PSR)).

It may seem callous to focus on the “long-term” effects of war while these horrific consequences of war are here and now. Nevertheless, long-term public health consequences of war need to be better examined if we are to prevent similar wars in the future (Weir 2015). To that end, here we report the results of our last samples from a growing war-zone.

Deciduous teeth of children from Iraq, Lebanon, and Iran can show a continuum of high to low war-related-exposures in children. Measurements of environmental samples in the areas of our interest are rare in the literature. Therefore, we deduce that a continuum of high to low war-related exposures can be detected in children of the selected areas based upon the knowledge of the number and length of wars fought in each country in modern times. We do know that Iraq continues to be the target of repeated bombings and military activity, that Lebanon has been the site for multiple wars, and that military activities have occurred in Lebanon intermittently up to 2016 (Haugbolle 2010). In contrast, Iran has been the site of only one war in modern times, which ended in 1988 (Hersh 1992). Our aim is to evaluate deciduous teeth for their suitability to serve as markers of prenatal exposures to neurotoxic heavy metals.

Metals are one of the main components of bombs, bullets, and other weaponry. Buncombe (2011) offers a historic account of the very large number of bombs and bullets that were dropped in the Middle East post-2003. Additionally, 1500 US military bases and facilities—with their associated toxic pollutants—have been erected in the Middle East since 2003 (Nazaryan 2014; Vine 2014). It has been suggested that US military bases are among the most polluting operations on earth (Nazaryan 2014; Broder 1990; Milmo 2014).

In Iraq, there are currently over 500 US military bases (Kennedy 2008; Vine 2014). Pollutants released from these bases have reportedly harmed human health (Institute of Medicine, IOM 2011). Metals are released in the environment in large quantities during and following wars, either by direct bombing or as a result of waste generated and released by military installations (IOM). Metals are persistent in the environment (Li et al. 2014), and their adverse effects on health—especially the health of sensitive populations (i.e., pregnant mothers, fetuses, growing children)—have been established (Parajuli et al. 2013; Grandjean and Landrigan 2014). Public exposure to war-related pollutants intensifies as wars become frequent and as the environmental release of waste associated with military bases increases. Metal exposures and toxicity are frequently reported in children, particularly those living in areas of protracted military attacks in the Middle East (Alsabbak et al. 2012; Jergovic et al. 2010; Savabieasfahani et al. 2015).

As prenatal exposures become more severe and common in war zones, the accurate measurement of those prenatal exposures becomes more urgent. The use of deciduous teeth, which originate in fetal life, as a biomarker of prenatal exposure, is worthwhile (Landrigan 2004) if we are to protect children from such exposures in the future. Teeth have been used as bio-indicators of environmental exposure to metals and to subsequent disease occurrence (Hare et al. 2011; Arora and Austin 2013). For example, Pb levels in primary teeth are accepted indicators of environmental Pb exposure in children and adolescents and have been linked to neurodevelopmental disorders (Arora et al. 2006).

Elemental bio-imaging with laser ablation-inductively coupled plasma-mass spectrometry (LA-ICP-MS) is a new method for imaging trace elements in tooth. Based on tooth LA-ICP-MS studies, deciduous teeth have been proposed as an effective past exposure biomarker that can objectively and precisely illustrate the intensity and timing of past exposures (Arora and Austin 2013). Fine-tuning of this method will significantly improve our ability to quantify children’s exposures at different life stages and will enhance the quality of risk assessment studies for children.

**Fig. 1:**
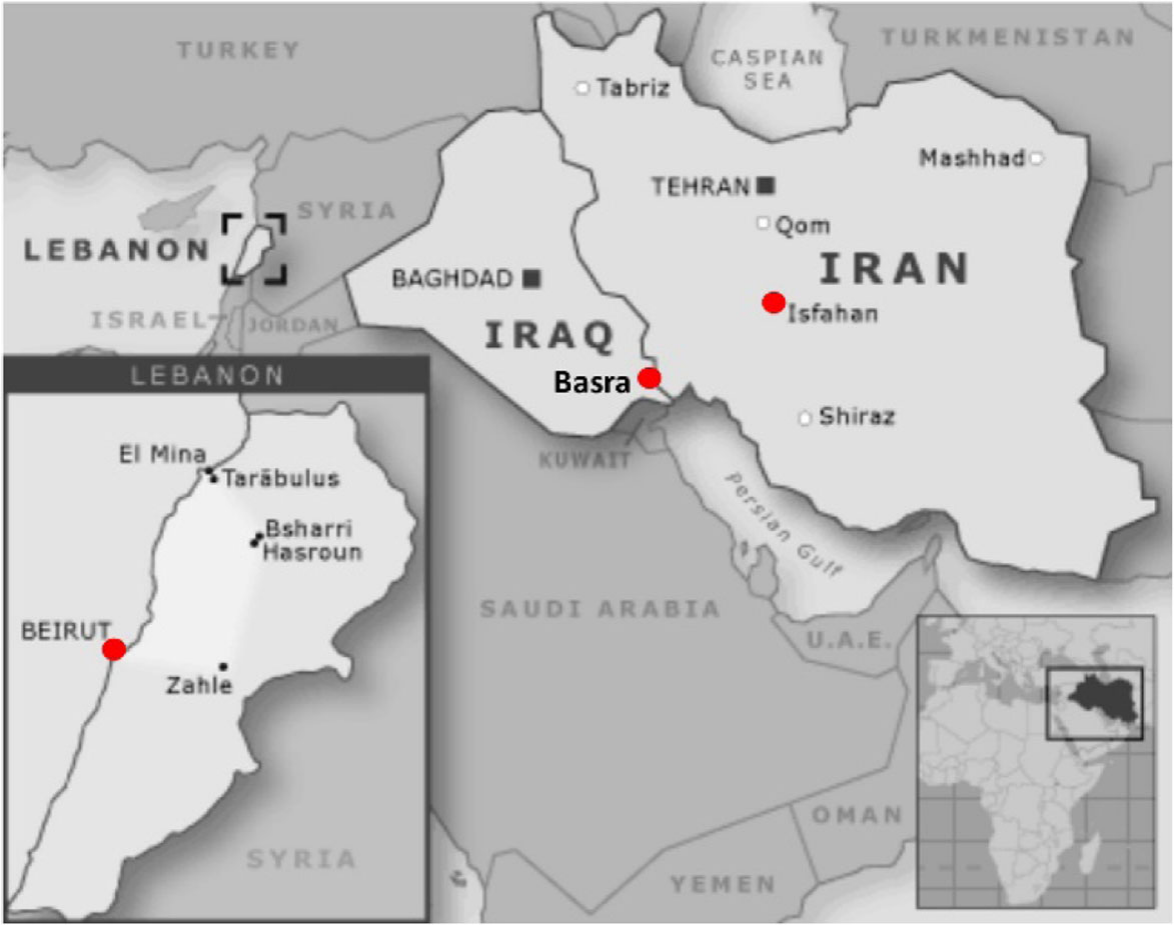
Map of the study area in the Middle East, Basra, Beirut, and Isfahan are marked in *red circles*

War-related metal pollution occurs in varying degrees in Iraq, Lebanon, and Iran (Fig. 1). Children’s deciduous teeth from these areas ought to bear the mark of these variable exposures.

**Fig. 2:**
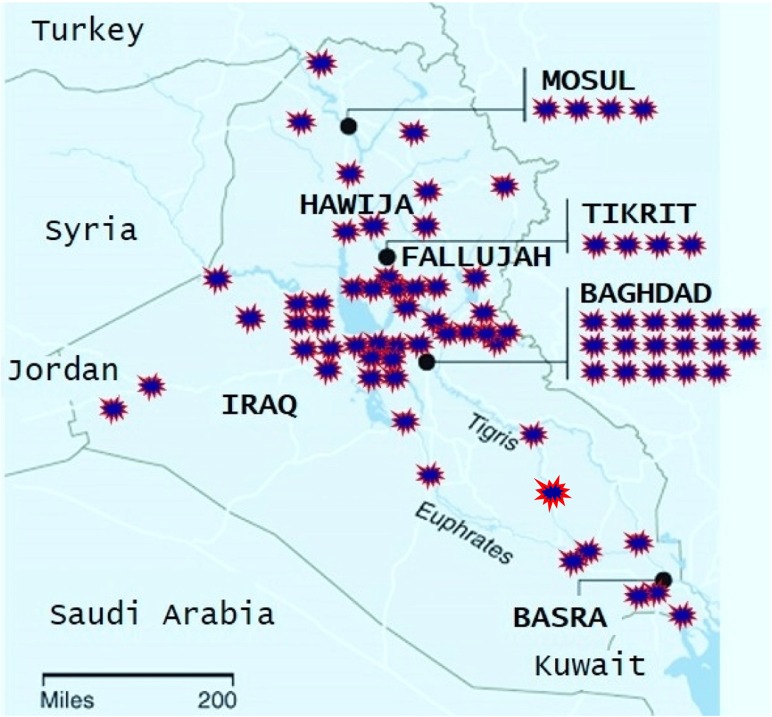
US military bases are indicated by . Basra is surrounded by military bases that have been continuously releasing war-related pollutants, including metals, into the Iraqi environment

Basra City, in Iraq, is surrounded by military bases (Fig. 2), and it has been the site of numerous military attacks. Beirut, in Lebanon, has seen a civil war and intermittent bombings and military attacks. Isfahan, a south central city in Iran, has not witnessed any military attacks in recent history. We therefore expected to see high, medium, and low metal exposures in the deciduous teeth of children from Basra, Beirut, and Isfahan, respectively. Elemental bio-imaging of these selected deciduous teeth can improve our knowledge of prenatal exposures to toxic metals.

## Material and methods

### Tooth donation and sample preparation

In April 2013, deciduous teeth were collected in two public clinics, one in Isfahan, Iran (*n* = 2; 9, and 12 years old; mean age = 10.5), and the other in Basra, Iraq (*n* = 3; 5, 8, and 14 years old; mean age = 9). A private clinic in Beirut, Lebanon, also collected deciduous teeth for this study (*n* = 4; 11, 13, 6, and 6 years old; mean age = 9). Prior to tooth collection, parents of the potential tooth donors were informed about our research interest. Parents were made fully aware of the way the children’s teeth will be used in this research. Samples of tooth used in this study were collected from children of those parents who gave their full consent to our proposed research. Parental consent forms were signed at the clinic in the presence of the licensed dentist. The children of Isfahan and Beirut were in good health but the deciduous teeth from Basra were donated by children who had survived various birth defects such as soft tissue anomalies and neural tube defects. They also lived in areas that had been bombed.

Iraqi children who donated teeth to this study confirmed that they lived in homes that were bombed (at least once) and also near a known bombed site. One of the Lebanese participants (the 12-year-old) lived in a home that was bombed and was also located near a known bombed site. Iranian children’s dwellings were not bombed, and they did not live near a known bombed site.

Tooth samples were cleaned of saliva, blood, and other tissue debris. Then those samples were dried at room temperature and wrapped in clean medical gauze. Each tooth was then placed in a clean autoclaved paper envelope. After closing the envelopes, identification information was pasted on each envelope. Closed envelopes were mailed, in batches, from each country, to the corresponding author. Iranian and Lebanese teeth were in better condition upon arrival, compared to Iraqi samples.

Longitudinal sections were made of teeth after resin embedding for Basra and Isfahan teeth. Deciduous teeth from Beirut were not embedded in resin for sectioning. The sections were polished with 1-μm diamond paste and washed in Milli-Q water prior to analysis.

## Image processing

Elemental images of ^7^Li, ^25^Mg, ^43^Ca, ^52^Cr, ^55^Mn, ^66^Zn, ^88^Sr, ^138^Ba, ^208^Pb, ^232^Th, and ^238^U in tooth were generated. Quantitative information is based on previously published single-point calibration (Hare et al. 2011).

Elemental maps were made of the sections using laser ablation-inductively coupled plasma-mass spectrometry (LA-ICP-MS). Elements scanned for were ^7^Li, ^25^Mg, ^43^Ca, ^52^Cr, ^55^Mn, ^66^Zn, ^88^Sr, ^138^Ba, ^208^Pb, ^232^Th, and ^238^U. ^43^Ca was used as an internal standard (all elements normalized to Ca). Laser parameters were chosen to yield pixel size of about 35 × 35 μm. The certified glass standard NIST 612 was used for semi-quantification of trace element levels in the samples.

## Laser ablation-inductively coupled plasma-mass spectrometry

Operating conditions for the optimized LA-ICP-MS system, including variable parameters, plasma power, argon gas flow rates, sample depth (and thereby residence time of analytes in the plasma), lens voltages for ion beam focusing, and integration times for the mass spectrometer, have been reported earlier (Hare et al. 2011).

## Results and discussion

**Fig. 3:**
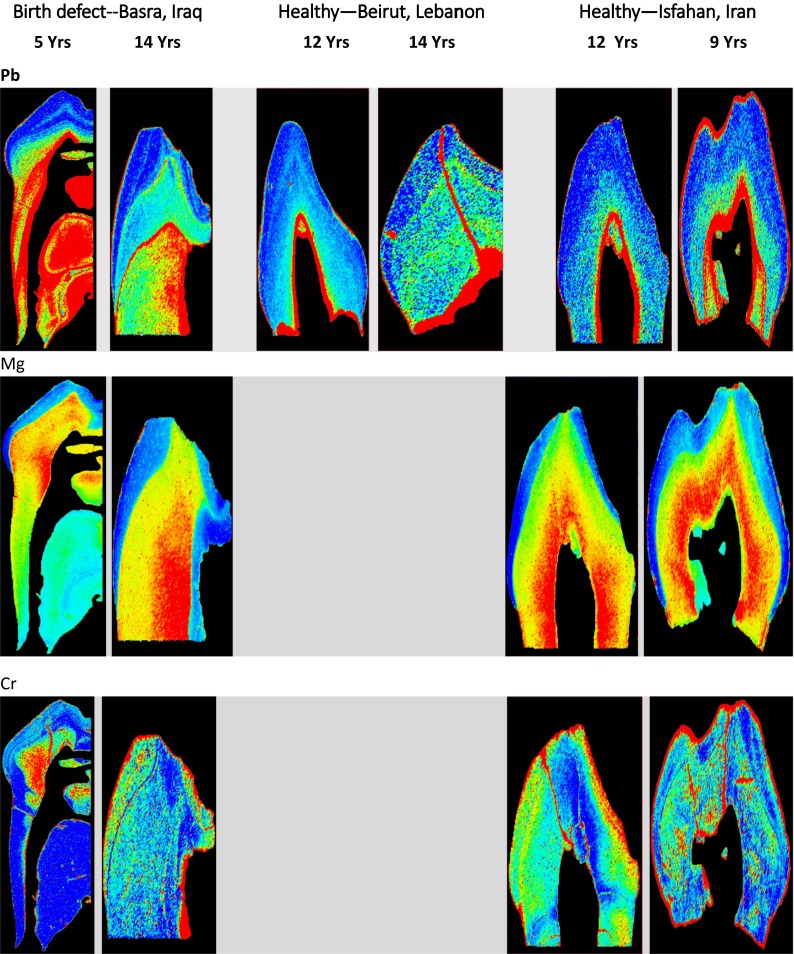

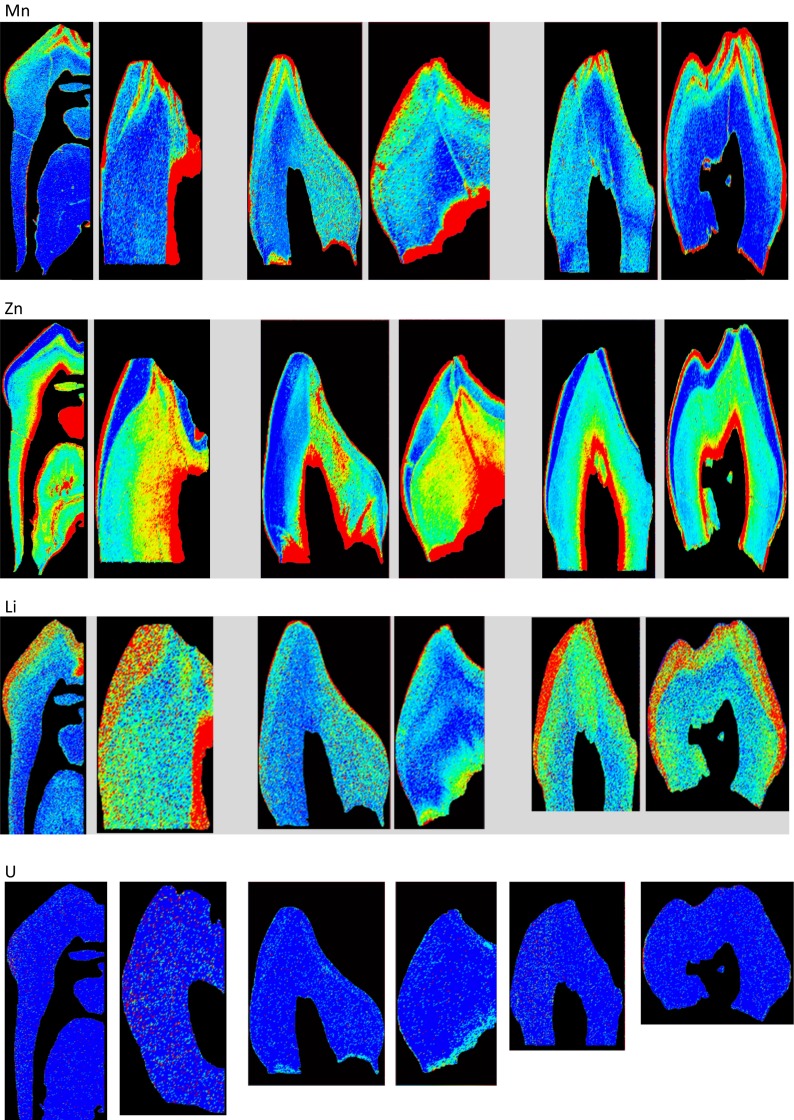
Lead (*Pb*), magnesium (*Mg*), chromium (*Cr*), manganese (*Mn*), zinc (*Zn*), lithium (*Li*), and uranium (*U*) in deciduous teeth of children from Iraq (high exposure to war pollutants), Lebanon (intermediate exposure to war pollutants), and Iran (low exposure to war pollutants)

Quantitative element maps of trace elements in deciduous teeth were created by the single-point calibration LA-ICP-MS method (Hare et al. 2011). Color index represents the range in metal levels from low (dark blue) to high (red) Fig. 3, Table 1.

**Table 1:** Range of metals in samples of tooth from Iraq, Lebanon, and Iran in ppm

Residence bombed	Country	Child’s age	^208^Pb/^43^Ca ppm	^25^Mg/^43^Ca ppm	^52^Cr/^43^Ca ppm	^55^Mn/^43^Ca ppm	^66^Zn/^43^Ca ppm	^7^Li/^43^Ca ppm	^232^Th/^43^Ca ppm	^238^U/^43^Ca ppm
Bombed	Iraq	5	0.17–0.87	94–540	1.03–5.2	0.10–0.52	1.8–4.5	0.022–0.08	0–0.2	0–0.13
Bombed	Iraq	8	1.9–48	288–1408	0.77–2.2	0.16–0.55	1.1–5.5	0.006–0.06	0–0.09	0–0.06
Bombed	Iraq	14	0.13–1.35	192–1408	0.77–1.65	0.22–1.1	1.1–5.0	0.011–0.08	0–0.09	0–0.06
Not bombed	Lebanon	7	0.034–0.34	Data unavailable	Data unavailable	0.24–1.6	8.0–400	0.007–0.15	0–0.1	0–0.1
Bombed	Lebanon	12	0.05–0.68	Data unavailable	Data unavailable	0.16–1.2	2.4–16	0.004–0.07	0–0.1	0–0.1
Not bombed	Lebanon	13	0.034–0.34	Data unavailable	Data unavailable	0.24–0.80	1.6–4.0	0.007–0.07	0–0.1	0–0.1
Not bombed	Lebanon	14	0.034–0.17	Data unavailable	Data unavailable	0.16–0.64	0.8–4.0	0.015–0.15	0–0.1	0–0.1
Not bombed	Iran	9	0.043–0.43	94–563	1.16–3.22	0.19–1.03	1.3–7.7	0.016–0.08	0–0.2	0–0.1
Not bombed	Iran	12	0.039–0.19	256–1664	0.44–1.43	0.11–0.55	1.1–5.5	0.017–0.08	0–0.09	0–0.06

Lead was consistently found in enamel and in dentine and in dentine-pulp junction (DPJ). Lead concentrations increase in the dentine when approaching the pulp cavity, and it remained high along DPJ. A narrow band of high Pb was evident on the surface of the enamel. The width of the band was variable. Lead appeared at similarly high levels in both the enamel surface and the DPJ. However, DPJ showed larger Pb deposits. Variation in Pb concentration was negligible across the band on the surface of the enamel and also in the DPJ. Maximum Pb levels were consistently detected in the Iraqi samples (1.9–48 ppm). The lowest Pb levels were found in the samples from Iran (0.039–0.19 ppm). Pb was highest in teeth from children with birth defects who donated their teeth from Basra, Iraq (mean 0.73–16.74 ^208^Pb/^43^Ca ppm, *n* = 3). Pb in healthy Lebanese and Iranian teeth were 0.038–0.382 ^208^Pb/^43^Ca ppm (*n* = 4), and 0.041–0.31 ^208^Pb/^43^Ca ppm (*n* = 2), respectively. Lead levels were similar in Lebanese and Iranian deciduous teeth. Deciduous teeth from Iraqi children with birth defects had remarkably higher levels of Pb. Two Iraqi teeth had four times more Pb, and one tooth had as much as 50 times more Pb than samples from Lebanon and Iran.

Elemental magnesium (Mg) images were created only for samples from Iraq and Iran. Mg consistently appeared in the dentine. Magnesium concentration markedly increased in the dentine when approaching the pulp cavity. The highest Mg levels were found in samples from Isfahan, Iran (256–1664 ^25^Mg/^43^Ca ppm).

Chromium (Cr) was highest in deciduous teeth from Iraq (1.03–5.2 ^52^Cr/^43^Ca ppm), and it appeared along the enamel surface and in the dentine region of all teeth. Cr in dentine appears to increase when approaching the pulp cavity and then decrease again near DPJ. Cr was also evident along a vertical line that started from the tooth surface and continued through the dentine, reaching the pulp.

Manganese (Mn) was highest in teeth from Beirut, Lebanon (0.24–1.6 ^55^Mn/^43^Ca ppm). Comparable levels of Mn existed in samples from Iraq and Iran. High Mn levels were detectable near the incisal tip in the region of the prenatally formed dentine. Mn was also evident in the form of a thin band on enamel surface, the width of the band varied.

Tooth zinc (Zn) levels were highest in Beirut, Lebanon. Maximum Zn concentration varied from 1.1–5.5 ppm to 8.0 to 400 ppm. In all samples, Zn concentrations in enamel were lower than in the dentine. Within the enamel, the highest Zn levels were seen at the outer edge with the inner enamel having very low concentrations. In the dentine, Zn was concentrated at the dentine-pulp margin and in the cervical dentine.

Uranium and thorium were not detected in any of the samples. Detection limits for U and Th are estimated to be in the ppb range for LA-ICP-MS analysis. Concentrations of U and Th in human teeth have been reported as <1 ppm.

Knowing that an estimated 1000 to 2000 metric tons of depleted uranium was fired during the 2003 war in Iraq (UNEP 2007 Annual Report), we had expected to detect uranium in deciduous teeth of children from Basra. However, surprisingly, U was undetectable in samples from Basra.

Our original hypothesis that increased war activity coincides with increased metal levels, including Pb, in deciduous teeth is confirmed by this research. Recent studies have used neonatal line as a reference point to determine prenatal or postnatal timing of exposure (Arora et al. 2014). Neonatal line is a histological feature formed in enamel and dentine at birth. Location of the prenatal line, which represented approximately 12–15 days of dentine deposition around birth, can provide a landmark to distinguish prenatally formed parts of teeth from those formed postnatally. Prenatal line appears as a distinct line in a light micrograph. The same study found that Pb levels in dentine formed at birth were significantly associated with cord blood Pb. This suggests that micro-spatial measurements of Pb in dentine can be used to show Pb exposure timing over the prenatal and early childhood periods and that secondary dentine has the potential to estimate long-term exposure up to the time the tooth is shed.

In Iraq today, an epidemic of birth defects is unfolding in many cities (Al-Sabbak et al. 2012). A variety of birth defects, some never seen before, are observed in hospitals routinely by researchers and physicians. Current knowledge is clear in that prenatal metal exposure can lead to birth defects. A comprehensive study of birth defects as related to the type of prenatal metal exposure (determined by the use of in deciduous teeth) can contribute to our understanding of how metals cause birth defects in humans. It may then become possible to answer questions regarding prenatal exposures to specific metals which are more frequently associated with certain birth defects.

Finally, it is important to mention that this current study concerns children's exposure to Pb during the military bombardment and occupation of Iraq. We present this article during an extraordinary period of public awareness concerning children’s Pb poisoning in Flint, Michigan. This Pb poisoning in Flint occurred at a time when the elected city government was effectively deposed by emergency state administrators. They eliminated Flint’s safe water source in favor of an inexpensive but hazardous water source. This has generated scholarly and mass media coverage, creating a global awareness of Pb poisoning and its dangers to the most vulnerable part of the population, namely children. An American Public Health association publication by Hanna-Attisha et al. clearly states the current knowledge about Pb poisoning in children and that Pb “… is a potent neurotoxin, and childhood Pb poisoning has an impact on many developmental and biological processes, most notably intelligence, behavior, and overall life achievement. With estimated societal costs in the billions, Pb poisoning has a disproportionate impact on low-income and minority children. When one considers the irreversible, life-altering, costly, and disparate impact of Pb exposure, primary prevention is necessary to eliminate exposure” (Hanna-Attisha et al. 2016).

## Conclusion

Our previous work in Iraqi cities confirm that, as in Flint, Iraqi children have been exposed to comparable Pb, as well as other neurotoxic metals, many in utero (Alsabbak et al. 2012; Savabieasfahani et al. 2015). Cleanup and preventive measures are critically needed in Iraqi cities which have endured a huge burden of contamination created by war.
